# Rethinking the Assessment of Arthrogenic Muscle Inhibition After ACL Reconstruction: Implications for Return-to-Sport Decision-Making—A Narrative Review

**DOI:** 10.3390/jcm14082633

**Published:** 2025-04-11

**Authors:** Florian Forelli, Ayrton Moiroux-Sahraoui, Jean Mazeas, Jonathan Dugernier, Adrien Cerrito

**Affiliations:** 1 Haute-Ecole Arc Santé, HES-SO University of Applied Sciences and Arts Western Switzerland, 2800 Delémont, Switzerland; jonathan.dugernier@he-arc.ch (J.D.); adrien.cerrito@he-arc.ch (A.C.); 2 Orthopaedic Surgery Department, Clinic of Domont, Ramsay Healthcare, @OrthoLab, 95330 Domont, France; ayrton.moirouxsahraoui@gmail.com (A.M.-S.); jeanmazeas@gmail.com (J.M.); 3 Société Française des Masseurs—Kinésithérapeutes du Sport Lab, 93380 Pierrefite sur Seine, France; 4 Orthosport Rehab Center, 95330 Domont, France

**Keywords:** arthrogenic muscle inhibition, anterior cruciate ligament reconstruction, neuromuscular recovery, return to sport, biomechanics

## Abstract

Arthrogenic muscle inhibition (AMI) is a neuromuscular impairment commonly observed following anterior cruciate ligament reconstruction (ACLR). This condition, characterized by persistent quadricep inhibition due to altered afferent feedback, significantly impacts neuromuscular recovery, delaying return to running and sport. Despite advancements in rehabilitation strategies, AMI may persist for months or even years after ACLR, leading to muscle strength asymmetries, altered biomechanics, and an increased risk of reinjury. The mechanisms underlying AMI involve both peripheral (joint effusion, mechanoreceptor dysfunction) and central (corticospinal inhibition, neuroplasticity alterations) components, which collectively hinder voluntary muscle activation and movement control. AMI alters gait mechanics, reduces knee stability, and promotes compensatory patterns that increase injury risk. Current return-to-sport protocols emphasize strength symmetry and functional performance but often neglect neuromuscular deficits. A comprehensive assessment integrating neuromuscular, biomechanical, and proprioceptive evaluations is needed at specific stages to optimize rehabilitation and minimize reinjury risk. Future research should explore targeted interventions such as neuromuscular stimulation, cognitive–motor training, and advanced gait analysis to mitigate AMI’s impact and facilitate a safer, more effective return to sport.

## 1. Introduction

Anterior cruciate ligament (ACL) injuries are among the most common and functionally limiting knee injuries, particularly in young and athletic populations [1]. They are frequently caused by non-contact mechanisms, such as sudden deceleration, pivoting, or awkward landings, and are often associated with concomitant damage to meniscal and cartilage structures [2,3]. Epidemiological studies show a higher prevalence in female athletes and pivoting sports such as soccer, basketball, and skiing [4].

Early and accurate diagnosis of ACL ruptures is crucial for planning treatment and rehabilitation. While magnetic resonance imaging (MRI) remains the gold standard for confirming ACL tears, clinical examination plays a critical role in the initial assessment [2]. Commonly used tests include the Lachman test, the anterior drawer test, and the pivot shift test [5]. Recently, the lever sign test has emerged as a promising diagnostic tool, showing high specificity and sensitivity across multiple clinical contexts [6]. Hesmerg et al. demonstrated its diagnostic accuracy in over 3000 observations, with pooled sensitivity and specificity values of 83% and 91%, respectively [6,7]. These findings were echoed in field studies, including assessments conducted in ski resorts.

Comparative studies such as those by Krakowski et al. and Makhmalbaf et al. have shown that clinical examination, when performed systematically, can approach the diagnostic accuracy of MRI for ACL and meniscal injuries [8,9]. These insights emphasize the importance of integrating both manual and imaging-based diagnostic tools into early ACL injury management.

ACL reconstruction (ACLR) is one of the most common orthopedic procedures performed on athletes and physically active individuals following an ACL rupture [10]. Despite advancements in surgical techniques and rehabilitation protocols, a significant proportion of patients experience persistent neuromuscular impairments that may delay or compromise their return to running (RTR) and return to sport (RTS) [11]. One of the primary factors contributing to these deficits is arthrogenic muscle inhibition (AMI)—a condition characterized by an involuntary decrease in muscle activation due to altered afferent feedback from the injured joint [12,13].

AMI is a neuromuscular protective response that occurs after joint trauma or surgery, reducing voluntary muscle activation [14,15,16]. It is particularly evident in the quadriceps, which play a crucial role in knee stability and dynamic control [17]. The persistence of AMI after ACLR can impede neuromuscular recovery, resulting in muscle weakness, abnormal gait mechanics, and compensatory movement patterns which may increase the risk of reinjury [18].

The underlying mechanisms of AMI involve both peripheral and central neurological alterations. Peripheral factors include joint effusion, altered mechanoreceptor signaling, and nociceptive input, which contribute to the inhibition of the alpha motor neurons innervating the quadriceps [19]. On a central level, AMI is associated with reduced corticospinal excitability and increased intracortical inhibition, further impairing voluntary muscle activation [20]. These neurophysiological changes can persist for months or even years after ACLR, significantly affecting functional performances and the ability to safely resume high-impact activities such as running and sports [15,21].

RTR and RTS are critical milestones in ACLR rehabilitation, often serving as a transitional phase between early rehabilitation and sport-specific training [22,23]. However, persistent AMI can delay or impair this transition by reducing quadricep strength, altering movement patterns, and negatively influencing dynamic knee stability [24]. These impairments have been linked to asymmetrical loading during running that may lead to increased stress on the reconstructed knee and contralateral limb, ultimately predisposing athletes to secondary ACL injuries [25,26].

Furthermore, AMI has been implicated in deficits in landing mechanics, single-leg stability, and reaction time, which are all essential components of RTR and RTS [27]. The failure to address AMI prior to RTR and RTS can result in suboptimal neuromuscular control, increasing the likelihood of reinjury and prolonging the rehabilitation timeline [23,28,29]. Despite its clinical significance, there is still, to our knowledge, no standardized approach to evaluate and manage AMI in RTR and RTS protocols, highlighting a gap in the existing rehabilitation framework [20].

Although several reviews have addressed quadricep strength recovery, RTS criteria, or neuromuscular control in patients with ACLR, few have focused specifically on AMI as a central barrier to rehabilitation [16,20]. The existing literature often treats AMI as a secondary phenomenon, rather than a primary contributor to delayed functional recovery and reinjury risk [12,19].

Moreover, current reviews typically lack an integrative perspective combining peripheral and central neurophysiological mechanisms with biomechanical, functional, and psychological implications [27,30].

Therefore, the rationale for this narrative review is to synthesize current evidence on AMI after ACLR, highlight unresolved issues regarding its assessment and management, and underscore the need for AMI-specific criteria in RTR and RTS decision making.

This review distinguishes itself by (1) consolidating recent findings on the central and peripheral mechanisms of AMI; (2) linking AMI to real-world performance impairments during RTR and RTS phases; and (3) proposing a multidimensional framework for assessment that incorporates neuromuscular, biomechanical, and psychological domains.

By addressing these underexplored areas, this work aims to bridge the gap between neurophysiological insights and practical rehabilitation strategies.

## 2. Methodology

This review follows a structured narrative approach, inspired by scoping review frameworks such as the Preferred Reporting Items for Systematic Reviews and Meta-Analyses extension for Scoping Reviews (PRISMA-ScR) guidelines, to ensure transparency and reproducibility, even though it does not fully adopt the formal structure of a systematic review.

### 2.1. Databases and Search Strategy

We conducted a literature search in PubMed, Web of Science, and Google Scholar from January 2000 to February 2024. The last search update was performed on 20 February 2024. The following search terms and Boolean combinations were used:

- (“arthrogenic muscle inhibition” OR “AMI”) AND (“ACL reconstruction” OR “anterior cruciate ligament”);
- (“quadriceps inhibition” OR “central activation failure”) AND (“neuromuscular control” OR “corticospinal excitability”);
- (“return to sport” OR “return to running”) AND (“biomechanics” OR “gait analysis”);
- (“functional performance” OR “rehabilitation outcomes”) AND (“ACL” OR “AMI”).

### 2.2. Inclusion and Exclusion Criteria

We included the following:

- Peer-reviewed articles;
- Works focused on AMI mechanisms, assessment, or rehabilitation following ACL injury or reconstruction;
- Studies reporting neuromuscular, biomechanical, or functional outcomes;
- Studies in English, published between 2010 and 2024, with a focus on recent developments (2022–2024).

We excluded the following:

- Conference abstracts without full-text availability;
- Case reports and expert opinion pieces lacking empirical data;
- Non-English publications.

### 2.3. Study Selection and Relevance

The initial screening was based on titles and abstracts. Full texts were then reviewed for relevance to the objectives of this narrative review. Additional references were identified through citation tracking of key systematic reviews and position statements.

## 3. Neurophysiological Mechanisms of AMI

AMI following ACLR is a neuromuscular impairment caused by altered sensory feedback and central motor dysfunction. It persists beyond the post-surgical period, delaying RTR and RTS by impairing muscle activation. Both peripheral and central nervous system alterations contribute to this deficit.

### 3.1. Central and Peripheral Contributions to AMI

One of the primary drivers of AMI is altered afferent feedback from damaged joint structures. The ACL contains mechanoreceptors that play a crucial role in proprioceptive feedback and neuromuscular control. When the ligament is ruptured and subsequently reconstructed, these mechanoreceptors are either lost or impaired, disrupting sensory input to the central nervous system [20]. This disruption affects motor output by increasing inhibitory reflex activity at the spinal level, reducing voluntary muscle activation [31]. Furthermore, joint trauma and surgical intervention often result in intra-articular effusion, which has been shown to further inhibit alpha motor neuron excitability and limit quadricep activation [14,15,16,32,33].

Beyond the peripheral level, AMI is also associated with dysfunctions in spinal and supraspinal pathways. Increased spinal reflex inhibition has been demonstrated in patients with ACLR, where alterations in Ia afferent feedback contribute to the reduced excitability of quadriceps’ motor neurons [17,32]. Suppression of the H-reflex, an indicator of spinal cord excitability, is commonly observed in individuals recovering from ACL injuries, further reinforcing the role of spinal inhibition in AMI [34]. At the supraspinal level, reductions in corticospinal excitability and increased intracortical inhibition have been documented using transcranial magnetic stimulation. These findings suggest that ACL injury and subsequent reconstruction lead to maladaptive neuroplasticity in the motor control regions of the brain, which further limits voluntary muscle activation [35].

### 3.2. Muscle Groups Affected

Quadricep inhibition is the main functional consequence of AMI. The vastus medialis and lateralis show reduced activation [31], impairing knee stability and movement efficiency. This leads to strength asymmetries that persist beyond rehabilitation, increasing injury risk and delaying recovery [32,33].

Although the quadriceps are the most affected, other muscle groups also experience neuromuscular changes after ACLR [36]. The hamstrings, which serve as secondary stabilizers of the knee, often exhibit increased co-contraction in response to quadricep inhibition. This compensatory mechanism, while potentially protective, can alter normal joint mechanics and contribute to inefficient movement patterns [37]. Additionally, dysfunction in the gluteal muscles, particularly the gluteus medius and gluteus maximus, has been observed in patients with ACLR [38]. These muscles play a critical role in controlling frontal plane knee stability, and their delayed activation may lead to increased knee valgus angles, a key risk factor for ACL reinjury [27]. Changes in neuromuscular recruitment patterns have also been noted in the gastrocnemii and core stabilizers, indicating that AMI extends beyond the knee joint and affects the entire kinetic chain involved in dynamic movements [39].

### 3.3. Factors Influencing AMI Persistence

Several factors contribute to the persistence of AMI, with joint effusion and pain being two of the most significant [40]. The presence of intra-articular swelling activates nociceptive pathways, leading to reflex inhibition of the quadriceps. Research has shown that even small amounts of joint effusion can cause a measurable decrease in voluntary quadricep activation, suggesting that early management of swelling is critical to preventing prolonged AMI [41].

Proprioceptive deficits also play a crucial role in maintaining AMI. After ACLR, mechanoreceptor dysfunctions lead to impaired joint position sensitivity, which negatively affects motor planning and execution. This disruption in sensory feedback contributes to altered movement patterns that persist even after strength deficits appear to be resolved [30]. Furthermore, neuromuscular disuse exacerbates AMI by reinforcing inhibitory pathways. Reduced activation of fast-twitch muscle fibers during prolonged inactivity leads to further decreases in neural drive to the quadriceps, prolonging functional impairments [18,31].

## 4. Clinical and Functional Manifestations

AMI plays a significant role in delaying and altering the RTR and RTS process following ACLR. Even after an apparent recovery, residual neuromuscular deficits persist, affecting muscle activation, movement mechanics, and injury risk. These impairments compromise performance, increase asymmetries, and contribute to a higher likelihood of reinjury, making AMI a crucial factor to consider in rehabilitation programs [14].

### 4.1. Neuromuscular Deficits Affecting RTR and RTS

One of the most critical consequences of AMI is reduced quadricep activation, which limits knee stability and functional performance during high-impact activities such as running and cutting movements [20]. Quadricep inhibition leads to strength asymmetries, which persist beyond the typical rehabilitation period, increasing the risk of compensatory movement strategies that may affect overall movement efficiency [18]. Deficits in voluntary quadricep activation have been reported even months after ACLR, demonstrating that AMI does not necessarily resolve naturally and must be specifically addressed through targeted rehabilitation [13,35].

In addition to strength loss, AMI also results in delayed neuromuscular response times, which impair knee joint stability during dynamic movements. Patients with ACLR exhibit longer delays in quadricep activation to an external stimulus, meaning their muscles take more time to respond to sudden changes in load or joint positioning [42]. This delay is particularly problematic in running and sports-related movements, where rapid neuromuscular control is essential for injury prevention. A slower muscle response time can lead to poor shock absorption, increasing joint stress and the likelihood of secondary injuries [27].

### 4.2. Gait Alterations Due to AMI

The persistence of AMI has significant consequences on gait mechanics, particularly during the RTR and RTS phase. Individuals recovering from ACLR commonly demonstrate changes in stride length, knee flexion angle, and ground reaction forces, all of which indicate an altered motor control strategy [43,44]. These patterns are often driven by quadricep inhibition and may result in increased reliance on proximal (hip) and distal (ankle) joints [22].

Such compensatory adaptations may paradoxically increase the risk of secondary injury. For instance, overloading the non-injured limb can predispose athletes to contralateral ACL tears, observed in up to 30% of return-to-sport cases [25]. In the long term, altered muscle recruitment and weight distribution may contribute to joint degeneration and early osteoarthritis [41].

### 4.3. Clinical and Functional Tests

Due to its impact on neuromuscular function, AMI is best detected through clinical and functional tests. Single-leg hop tests assess limb symmetry, stability, and neuromuscular control. Patients with ACLR with persistent AMI often show shorter hop distances and greater landing variability, indicating quadricep strength deficits and impaired shock absorption [24].

In addition to functional hop tests, isokinetic strength testing is a key assessment tool for identifying persistent quadricep weakness and activation deficits. Even when patients demonstrate seemingly adequate functional performance, isokinetic assessments frequently reveal asymmetries in torque production, particularly at high contraction speeds [45]. This underscores the importance of integrating strength-based testing into return-to-sport decision making, as traditional functional assessments alone may fail to detect underlying AMI-related impairments.

Advanced biomechanical assessments, including motion analysis and force plate testing, have also been used to detect subtle compensatory strategies linked to AMI. These tools allow clinicians to quantify joint kinematics, kinetics, and neuromuscular activation patterns in real time, providing valuable insights into movement quality and readiness for sport-specific tasks [30]. The combination of functional, strength-based, and biomechanical assessments is crucial for accurately identifying AMI-related deficits and ensuring a safe return to activity.

### 4.4. Correlation Between Persistent AMI and Reinjury Risk

One of the most concerning implications of AMI is its direct link to increased reinjury risk, particularly in the context of non-contact ACL reinjuries. Persistent quadricep inhibition leads to altered knee loading mechanics, which can predispose athletes to excessive stress on the ACL graft and surrounding structures [37]. Impaired biomechanics when returning to sport also usually lead to a higher risk of kinesiophobia and fear, which, by themselves, lead to modifying in-game motor patterns and increasing injury risks. This biomechanical instability increases the likelihood of experiencing a graft rupture or contralateral ACL tear, particularly in athletes who return to sport before fully restoring quadricep function [25].

Research has shown that quadricep strength symmetry is a critical factor in reducing reinjury risk. Athletes with residual quadricep strength asymmetry greater than 10% are at significantly higher risk of sustaining a secondary ACL injury within two years of returning to sport [28]. Additionally, deficits in neuromuscular control and dynamic stability—both of which are influenced by AMI—are among the strongest predictors of reinjury [14]. These findings emphasize the need for objective neuromuscular assessments to guide return-to-sport decisions, rather than relying solely on time-based criteria.

Beyond ACL reinjury, the long-term presence of AMI has been associated with joint degeneration and post-traumatic osteoarthritis. Altered loading mechanics resulting from chronic quadricep inhibition can accelerate the breakdown of articular cartilage, increasing the risk of early-onset knee osteoarthritis in young athletes after ACLR [41]. This highlights the long-term consequences of unresolved AMI, reinforcing the importance of comprehensive rehabilitation strategies aimed at restoring full neuromuscular function before returning to high-impact activities.

By influencing muscle activation, movement quality, and knee stability, AMI significantly delays RTR and RTS after ACLR. Addressing these neuromuscular deficits through comprehensive assessment and targeted rehabilitation is essential to optimize the rehabilitation process.

## 5. Assessment of AMI

An accurate assessment of AMI is essential for determining its impact on RTR and RTS after ACLR. Since AMI affects multiple neuromuscular components, a comprehensive evaluation approach is required, integrating electrophysiological, biomechanical, functional, and psychological assessments. Identifying AMI-related deficits through these tools is crucial for guiding rehabilitation strategies and minimizing reinjury risk [20].

### 5.1. Electromyography Analysis

Electromyography (EMG) is a widely used tool to quantify muscle activation deficits associated with AMI. The central activation ratio, obtained through superimposed burst techniques, is an important EMG-derived measure of voluntary activation deficits in the quadriceps after ACLR [20]. A reduced central activation ratio indicates persistent quadricep inhibition, which is a key characteristic of AMI.

Altered muscle recruitment patterns can also be assessed through surface EMG. Patients with ACLR commonly demonstrate delayed onset of quadricep activation, reduced amplitude of muscle firing, and compensatory co-contraction of the hamstrings and gastrocnemii [17]. These maladaptive neuromuscular strategies can impair dynamic knee stability, negatively affecting running mechanics and sport performance [18].

### 5.2. Isokinetic and Isometric Strength Testing

Isokinetic and isometric strength testing are considered gold standards for assessing quadricep inhibition and strength asymmetries in individuals with ACLR. Isokinetic dynamometry allows for the objective quantification of torque production, revealing deficits in maximal voluntary contraction and peak torque asymmetries [24]. Even in athletes who meet RTS criteria, residual strength deficits often persist, emphasizing the importance of continued monitoring [25].

Isometric testing, particularly at low knee flexion angles (e.g., 30° or 60° of knee flexion), has been used to assess quadricep activation deficits in individuals affected by AMI. Reduced force production during sustained contractions is indicative of persistent inhibitory mechanisms, which can limit performance in dynamic movements such as running and cutting [30].

### 5.3. Reflex and Corticospinal Excitability Tests

Since AMI involves both spinal and supraspinal dysfunctions, assessing reflex excitability provides valuable insight into its neurophysiological mechanisms. H-reflex testing, a measure of spinal reflex inhibition, has consistently demonstrated diminished excitability in patients with ACLR, suggesting increased inhibitory control over motor neurons [35].

At the cortical level, transcranial magnetic stimulation is used to assess corticospinal excitability and intracortical inhibition. Patients with ACLR frequently exhibit increased intracortical inhibition and reduced corticospinal drive to the quadriceps, reinforcing the role of neuroplastic alterations in AMI persistence [44]. These findings highlight the necessity of central neuromuscular retraining in ACLR rehabilitation programs.

### 5.4. Joint Effusion and Sensory Deficit Assessment

Joint effusion is a major peripheral contributor to AMI, and its presence can be evaluated through ultrasound imaging to quantify intra-articular swelling. Even small amounts of effusion have been linked to significant reductions in quadricep activation [41].

Sensory deficits, including impaired proprioception and kinesthetic awareness, are assessed through joint position sense tests and force reproduction tasks. Patients with ACLR often demonstrate reduced accuracy in detecting joint angle changes, which may contribute to movement asymmetries during running and sport-specific activities [30].

### 5.5. Functional and Performance-Based Evaluations

#### 5.5.1. Single-Leg Hop and Drop-Jump Tests

Functional hop tests are widely used to evaluate kinetic asymmetries and neuromuscular control impairments in patients with ACLR. The single-leg hop test provides an objective measure of the limb symmetry index, which is crucial for determining readiness to RTS [24]. Deficits in hop distance, increased landing variability, and reduced knee flexion angles during landing are commonly observed in individuals affected by AMI.

Similarly, the drop-jump test is valuable for assessing dynamic neuromuscular control. Patients with ACLR often exhibit increased knee valgus angles and reduced knee flexion moments, indicating compensatory strategies due to quadricep inhibition [39]. These alterations may predispose athletes to secondary injuries, emphasizing the importance of functional performance testing in ACL rehabilitation.

#### 5.5.2. Gait and Running Biomechanics’ Analysis

Gait analysis serves as an objective tool to quantify the biomechanical consequences of AMI. Using motion capture systems, force platforms, and instrumented treadmills, clinicians can measure spatiotemporal parameters such as stride length, stance-phase knee flexion angles, and ground reaction forces [46,47].

These technologies help identify persistent asymmetries and altered joint loadings that may not be visible through clinical observation alone. Gait analysis is particularly useful for monitoring progression through rehabilitation and informing return-to-sport readiness [22].

#### 5.5.3. Fatigue-Resistant Strength and Activation Tests

Given that AMI persists under fatigued conditions, sustained contraction tests are used to assess neuromuscular endurance. Prolonged isometric holds and repeated knee extension exercises have been shown to exacerbate quadricep inhibition, further highlighting the impact of AMI on performance sustainability [20]. Identifying fatigue-induced neuromuscular deficits is crucial for designing sport-specific rehabilitation programs.

#### 5.5.4. Patient-Reported Outcome Measures (PROMs)

Psychological factors significantly influence RTR and RTS readiness. The Tampa Scale of Kinesiophobia (TSK-11) is frequently used to assess fear of movement and reinjury, both of which can contribute to compensatory movement strategies and prolonged AMI [25].

Self-reported confidence in running and sport-specific activities is also evaluated using the ACL Return to Sport after Injury (ACL-RSI) questionnaire, which measures emotional readiness, performance confidence, and risk perception [28].

#### 5.5.5. Cognitive–Motor Integration Tests

Cognitive–motor function is increasingly recognized as a key component of neuromuscular recovery. Dual-task performance assessments, which require patients to perform motor tasks while engaging in cognitive challenges (e.g., reaction time tasks), have revealed altered cortical processing and motor planning deficits in individuals with ACLR [27,39]. These findings underscore the importance of cognitive–motor training in post-ACLR rehabilitation programs.

By integrating neuromuscular, biomechanical, functional, and psychological assessments, clinicians can identify the presence and persistence of AMI, tailor rehabilitation strategies, and ensure a safe and efficient transition back to running and sport-specific activities.

### 5.6. Empirical Evidence Supporting AMI Assessment

Beyond theoretical models, several empirical studies provide quantifiable evidence of AMI’s impact on neuromuscular control and function following ACLR. These studies have employed diverse methodologies—ranging from electromyography and isokinetic dynamometry to transcranial magnetic stimulation and 3D motion analysis—to objectively measure the extent and persistence of AMI-related impairments. Results consistently show reduced central activation of the quadriceps, strength asymmetries exceeding clinical thresholds, and altered cortical excitability. Functional outcomes, such as gait symmetry and ground reaction forces, are also measurably affected.

Table 1 provides a synthesis of these key findings, including effect sizes and statistical significance where available. These data underscore the importance of integrating objective neuromuscular assessments into return-to-sport decisions, rather than relying solely on subjective or time-based criteria.

## 6. Rehabilitation Strategies and Clinical Implications

AMI remains a significant barrier to full neuromuscular recovery following ACLR. Despite advancements in rehabilitation protocols, the persistence of AMI continues to compromise functional performance, delay RTR and RTS, and increase reinjury risk. Future research and clinical applications should focus on individualized assessment protocols, technological advancements, and improved clinical translation to optimize outcomes and reduce the likelihood of long-term neuromuscular impairments.

### 6.1. Need for Individualized Assessment Protocols

A key challenge in AMI management is the lack of personalized assessment protocols considering its severity and duration. Rehabilitation often relies on general strength and functional criteria without assessing neurophysiological deficits [20]. Due to AMI’s variability, tailored evaluations are essential to optimize rehabilitation.

A more patient-centered approach should integrate objective neuromuscular assessments (e.g., electromyography, isokinetic testing, H-reflex analysis) with functional performance metrics (e.g., single-leg hop tests, gait analysis) to ensure a comprehensive evaluation of AMI severity [30]. Moreover, clinicians should track AMI progression over time, adjusting rehabilitation interventions based on individualized neuromuscular recovery patterns rather than predetermined timeframes. Establishing personalized return-to-activity benchmarks could enhance the safety and efficiency of RTR and RTS decisions.

### 6.2. Advancements in Technology for AMI Assessment

Recent technological advancements present new opportunities for AMI assessment and rehabilitation. Wearable sensors and real-time gait analysis systems offer a promising avenue for the continuous monitoring of movement patterns and muscle activation deficits during rehabilitation [27]. These technologies provide immediate biofeedback, allowing clinicians to detect compensatory movement strategies and adjust interventions accordingly.

Artificial intelligence (AI) and machine learning are also being explored to enhance AMI detection and rehabilitation outcomes. AI-assisted gait analysis can identify subtle biomechanical abnormalities that may not be visible through traditional assessment methods [23]. Additionally, AI-driven rehabilitation platforms can provide automated neuromuscular feedback, guiding patients through progressive training regimens designed to restore normal motor control and minimize reinjury risk [37].

Another emerging technology in AMI management is neurostimulation therapy, including transcranial magnetic stimulation and functional electrical stimulation. These modalities have demonstrated potential in modulating corticospinal excitability, reducing intracortical inhibition, and enhancing voluntary muscle activation in patients with ACLR [35,49]. Future research should further explore the integration of neurostimulation techniques into conventional rehabilitation programs to accelerate AMI resolution and facilitate a smoother return to dynamic activities.

### 6.3. Challenges in AMI Research and Clinical Translation

Despite the growing body of research on AMI, several gaps remain in understanding its long-term effects on functional performance and reinjury risk. One key challenge is determining the time course of AMI recovery—while some patients experience the gradual resolution of neuromuscular inhibition, others exhibit persistent deficits that extend for years after ACLR [50]. Identifying the factors contributing to prolonged AMI is essential for developing targeted interventions and optimizing rehabilitation strategies.

Another challenge in AMI research is the limited clinical translation of neurophysiological findings. While studies using H-reflex, corticospinal excitability, and EMG analysis provide valuable insights into AMI mechanisms, these assessments are not commonly used in clinical settings due to their complexity and resource requirements [24]. Bridging the gap between laboratory research and real-world rehabilitation will require the development of clinically applicable assessment tools that provide rapid and practical insights into AMI status.

Additionally, longitudinal studies are needed to determine the relationship between persistent AMI and secondary injuries. While existing research suggests that quadricep inhibition and gait asymmetries contribute to contralateral ACL tears, more evidence is required to establish specific AMI-related risk factors for reinjury [25]. Future studies should focus on long-term monitoring of patients with ACLR, assessing neuromuscular function over several years to better understand AMI’s contribution to post-traumatic osteoarthritis and chronic instability.

### 6.4. Practical Recommendations for Clinicians and Researchers

To effectively address AMI in patients with ACLR, clinicians and researchers should prioritize integrating AMI-focused assessments into standard RTR and RTS criteria. Current return-to-sport guidelines primarily emphasize strength and functional symmetry, yet neuromuscular activation and central motor control are often overlooked [15]. Expanding RTR and RTS protocols to include neuromuscular assessments could significantly improve injury prevention strategies and enhance long-term knee health.

Clinicians should incorporate multi-modal AMI evaluations, combining neuromuscular, biomechanical, and psychological assessments to capture the full scope of impairment [22]. Objective markers of quadricep activation (e.g., central activation ratio, EMG patterns) should be used alongside functional movement tests (e.g., gait analysis, hop performance) to guide rehabilitation progression. Additionally, psychological screening tools, such as the Tampa Scale of Kinesiophobia (TSK-11), should be included in RTR and RTS decision making, as fear of reinjury can exacerbate AMI-related compensations [28].

From a research perspective, future studies should explore the efficacy of targeted interventions for AMI resolution, including cognitive–motor retraining, neuromuscular stimulation, and motor imagery techniques [27]. Investigating the role of fatigue in AMI persistence and evaluating the real-world applications of AI-based rehabilitation programs will also be critical for advancing clinical practice.

By prioritizing individualized assessment, technological innovation, and interdisciplinary collaboration, clinicians and researchers can enhance AMI detection, rehabilitation outcomes, and RTR and RTS safety [51]. Implementing these strategies will contribute to improved recovery trajectories, reduced reinjury rates, and enhanced long-term athletic performance in patients with ACLR.

### 6.5. Structured Rehabilitation Framework Targeting AMI

Interventions should be tailored to the patient’s progression and integrated into a comprehensive rehabilitation plan. Early-phase treatments such as neuromuscular electrical stimulation (NMES) and cryotherapy are primarily aimed at mitigating spinal reflex inhibition. As recovery progresses, mid-phase strategies like eccentric loading and blood flow restriction (BFR) target central drive and muscle hypertrophy [51]. Finally, late-stage interventions focus on cortical engagement and dynamic control through dual-task and sensorimotor training (Table 2).

**Figure 1:**
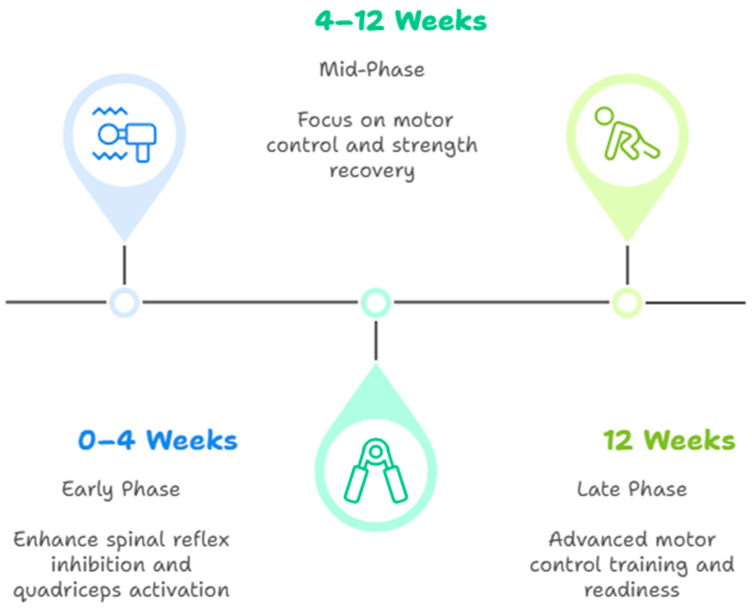
Optimizing rehabilitation with AMI-targeted interventions.

The effectiveness of AMI-targeted interventions can be better understood when organized by rehabilitation phase, with quantitative outcomes and evidence levels clarifying their clinical utility (Figure 1).

In the early phase (0–4 weeks), the primary goal is to enhance spinal reflex inhibition and quadricep activation. NMES, applied at 50–75 Hz for 20 min, five times per week, resulted in a 16% improvement in the central activation ratio (CAR from 0.80 to 0.93; *p* < 0.01) in a randomized controlled trial (Level I) [11]. Additionally, cryotherapy reduced pain and joint effusion, contributing to enhanced voluntary contraction (Level II) [5].

In the mid-phase (4–12 weeks), the focus shifts to motor control and strength recovery. BFR training, performed 2–3 times per week using low-load resistance protocols, has been shown to yield quadricep strength gains of up to 25% over 6–8 weeks (Level II) [5]. Eccentric training also improved EMG amplitude and muscle recruitment quality, with significant neuromuscular adaptation observed (Level II) [28].

In the late phase (>12 weeks), advanced motor control training is emphasized. Dual-task training and visuomotor biofeedback have been associated with up to a 15% improvement in limb symmetry index and better quality of movement during sport-specific tasks (Level III) [26]. Transcranial magnetic stimulation has shown a significant reduction in short-interval intracortical inhibition and an increase in motor-evoked potentials, indicating enhanced corticospinal excitability (*p* < 0.05; Cohen’s *d* = 0.80), although data are limited to exploratory controlled studies (Level II) [24]. Other study has shown that anodal transcranial direct-current stimulation is effective in reducing maladaptive quadricep inhibition and enhancing facilitation after ACLR, thereby targeting key neurophysiological mechanisms involved in AMI [52].

Some AMI-targeted protocols, combining neuromuscular and cortical approaches, have led to accelerated RTR and RTS readiness, with functional symmetry (e.g., hop tests) achieved approximately 2.5 months earlier compared to conventional rehabilitation [9]. The clinical efficacy of future studies should confirm these results and further quantify optimal dosing parameters and long-term outcomes.

### 6.6. Alignment with International Guidelines on Return to Sport

Several professional societies and expert groups have published structured recommendations to guide return to sport following anterior cruciate ligament reconstruction. The consensus from the Panther Symposium, published in 2016, proposes a multifactorial approach including at least 90% limb symmetry in quadricep strength, performance on hop tests, psychological readiness evaluated with validated tools such as the Anterior Cruciate Ligament–Return to Sport after Injury scale (≥56), and the ability to maintain movement quality under fatigue [53]. The American Orthopaedic Society for Sports Medicine, in agreement with the Multicenter Orthopaedic Outcomes Network group, similarly promotes an individualized, criteria-based model incorporating isokinetic strength testing, single-leg hop performance, biomechanical analysis of movements such as the drop vertical jump, and psychological evaluation using validated instruments such as the Tampa Scale of Kinesiophobia [54,55]. The Royal Dutch Society for Physical Therapy has developed a six-phase rehabilitation framework with specific performance benchmarks. Its criteria for return to sport include at least 90% quadricep strength symmetry, at least 95% symmetry on three out of four single-leg hop tests, and a psychological readiness score of at least 56 on the Anterior Cruciate Ligament–Return to Sport after Injury scale [56]. The International Society of Arthroscopy, Knee Surgery and Orthopaedic Sports Medicine also supports a criteria-based decision-making process, placing emphasis on the restoration of knee joint stability, neuromuscular control, and sport-specific confidence before returning to pivoting or contact sports [57]. In contrast, the European Society of Sports Traumatology, Knee Surgery and Arthroscopy has not yet published formal guidelines specifically addressing return to sport following ACLR. While certain opinion papers discuss long-term recovery and reinjury prevention, there is currently no official consensus defining objective performance thresholds [58]. This narrative review builds on the existing frameworks by highlighting the central role of AMI, a physiological factor which may delay or limit the ability to achieve established RTS criteria. Incorporating specific assessments may help refine clinical decision making and improve the safety and precision of RTS protocols.

## 7. Future Directions

To move beyond descriptive insights, future research on AMI should adopt more rigorous and translational methodologies. While AMI is well established as a short- to mid-term neuromuscular deficit after ACLR, its long-term consequences remain poorly understood, particularly in relation to post-traumatic osteoarthritis [27] and secondary ACL injuries [16,29].

First, there is a clear need for randomized controlled trials evaluating the efficacy, optimal timing, and dosage of targeted AMI interventions. These trials should include direct comparisons between modalities such as NMES, eccentric training, blood flow restriction, and cognitive–motor integration. Trials should incorporate quantifiable neuromuscular outcomes, including central activation ratio, quadricep symmetry index, corticospinal excitability, and gait symmetry, and follow participants through return-to-sport and reinjury timelines.

Second, prospective longitudinal cohort studies are necessary to investigate the role of unresolved AMI in the development of post-traumatic osteoarthritis. Persistent quadricep inhibition may lead to chronic joint instability, altered loading patterns, and cartilage degradation, accelerating degenerative changes. Future studies should include serial biomechanical assessments, quantitative MRI markers (e.g., T2 mapping), and functional testing to explore this potential pathway over multi-year follow-up.

Third, studies should examine the association between AMI and the risk of secondary ACL injuries, particularly in populations with neuromuscular asymmetries at the time of return to play. Integrating wearable technology and remote monitoring tools may allow better tracking of neuromuscular readiness and limb loading outside laboratory settings.

Finally, subgroup analyses are essential to understand how AMI manifests and responds to intervention in specific populations—such as adolescents, female athletes, or individuals with previous contralateral ACL injuries—and whether tailored rehabilitation strategies are needed for these higher-risk groups.

By combining controlled interventional studies with long-term follow-up designs, future research can help clarify not only how to treat AMI effectively, but also how to prevent its downstream consequences on joint health and injury recurrence.

## 8. Conclusions

AMI is a persistent and multifactorial neuromuscular impairment that significantly influences recovery following ACLR. Its effects extend beyond the acute phase, contributing to strength asymmetries, altered movement mechanics, and potentially increased risk of reinjury or long-term joint degeneration.

This narrative review has synthesized the central and peripheral mechanisms underlying AMI, its impact on neuromuscular performance, and its role in delaying RTS readiness. It also outlined a structured, phase-based rehabilitation framework, integrating evidence-based interventions such as neuromuscular electrical stimulation, eccentric exercise, and cognitive–motor strategies.

Importantly, there is a pressing need for future research to move beyond descriptive studies. High-quality randomized controlled trials should investigate the effectiveness, dosage, and optimal timing of these interventions. Longitudinal cohort studies are also essential to assess AMI’s potential contribution to post-traumatic osteoarthritis and secondary ACL injuries, especially in populations with persistent quadricep inhibition and neuromechanical asymmetries.

By addressing these gaps, future work can help define objective, AMI-informed criteria for return to sport and mitigate the long-term consequences of ACL injury.

## Figures and Tables

**Table 1:** Key quantitative findings on arthrogenic muscle inhibition after ACLR.

Study	Assessment Tool	Main Findings	Quantitative Data	Statistical Data
Lepley and Palmieri-Smith (2015) [20]	EMG—CAR	Reduced voluntary quadricep activation	CAR: 0.82 (injured) vs. 0.94 (uninvolved)	*p* = 0.002
Kuenze et al. (2015) [18]	Isokinetic dynamometry	Quadricep torque asymmetry at 6 months	Symmetry index ~73%	*p* < 0.001; Cohen’s d = 1.02
Pietrosimone et al. (2013) [35]	TMS	↑ intracortical inhibition (SICI), ↓ excitability	↓ MEP, ↑ SICI	*p* < 0.05; Cohen’s d = 0.80
Pamukoff et al. (2018) [48]	3D gait + force plate	↓ knee flexion angle, ↓ GRF	7–12° ↓ flexion; ↓ GRF ~10–15%	*p* < 0.01
Hart et al. (2010) [17]	EMG	Quadricep inhibition across conditions	18–25% ↓ EMG amplitude	Not quantified (review)
Pietrosimone et al. (2015) [49]	TMS + strength	Corticospinal predictors of quad strength	Positive correlation (r = 0.47)	*p* = 0.006
Baumeister et al. (2011) [42]	EEG—force control	Altered electrocortical patterns	Delayed theta-band response	*p* < 0.05
Büttner et al. (2024) [43]	Bilateral gait analysis	Asymmetry in loading and stride	↓ step length and GRF on involved side	*p* < 0.05

Abbreviations: EMG, electromyography; CAR, central activation ratio; TMS, transcranial magnetic stimulation; MEP, motor evoked potential; SICI, short-interval intracortical inhibition; GRF, ground reaction force; EEG, electroencephalography; ACLR, anterior cruciate ligament reconstruction; and AMI, arthrogenic muscle inhibition. ↑ increase and ↓ decrease.

**Table 2:** Phase-based rehabilitation strategies to address AMI.

Phase	Objective	Interventions	Frequency/Duration	Evidence/Effectiveness
Early (0–4 weeks)	Reactivate quadriceps, reduce reflex inhibition	NMES, cryotherapy, joint mobilization, and visual feedback	NMES: 5×/week, 20 min, 50–75 Hz; ice: 15 min post-ex	↑ quadricep activation [11], ↓ pain [5]
Mid (4–12 weeks)	Improve volitional contraction, restore neuromuscular control	Eccentric exercise, blood flow restriction, and motor imagery	BFR: 2–3×/week, 30/15/15/15 reps; eccentric: 3×/week	↑ strength gains [5], ↑ EMG [28]
Late (>12 weeks)	Reintegrate cognitive-motor control, prep for RTS	Dual-task training, perturbation, and sport-specific drills	2–3×/week, 30–60 min	↑ cortical reorganization [26], ↑ movement quality [14]

Abbreviations: NMES, neuromuscular electrical stimulation; BFR, blood flow restriction; RTS, return to sport; and EMG, electromyography. ↑ increase and ↓ decrease.

## Data Availability

Not applicable.
